# Not All Green Space Is Created Equal: Biodiversity Predicts Psychological Restorative Benefits From Urban Green Space

**DOI:** 10.3389/fpsyg.2018.02320

**Published:** 2018-11-27

**Authors:** Emma Wood, Alice Harsant, Martin Dallimer, Anna Cronin de Chavez, Rosemary R. C. McEachan, Christopher Hassall

**Affiliations:** ^1^School of Biology, Faculty of Biological Sciences, University of Leeds, Leeds, United Kingdom; ^2^Sustainability Research Institute, School of Earth and Environment, University of Leeds, Leeds, United Kingdom; ^3^Bradford Institute for Health Research, Bradford Teaching Hospitals NHS Foundation Trust, Bradford, United Kingdom

**Keywords:** green space, psychological restoration, biodiversity, park, urban, city, birds, plants

## Abstract

Contemporary epidemiological methods testing the associations between green space and psychological well-being treat all vegetation cover as equal. However, there is very good reason to expect that variations in ecological “quality” (number of species, integrity of ecological processes) may influence the link between access to green space and benefits to human health and well-being. We test the relationship between green space quality and restorative benefit in an inner city urban population in Bradford, United Kingdom. We selected 12 urban parks for study where we carried out botanical and faunal surveys to quantify biodiversity and assessed the site facilities of the green space (cleanliness, provision of amenities). We also conducted 128 surveys with park users to quantify psychological restoration based on four self-reported measure of general restoration, attention-grabbing distractions, being away from everyday life, and site preference. We present three key results. First, there is a positive association between site facilities and biodiversity. Second, restorative benefit is predicted by biodiversity, which explained 43% of the variance in restorative benefit across the parks, with minimal input from other variables. Third, the benefits accrued through access to green space were unrelated to age, gender, and ethnic background. The results add to a small but growing body of evidence that emphasize the role of nature in contributing to the well-being of urban populations and, hence, the need to consider biodiversity in the design of landscapes that enhance multiple ecosystem services.

## Introduction

Urban green spaces have been shown to improve health and well-being through conferring a number of ecosystem services (Scott et al., 2016) including buffering noise pollution (Margaritis and Kang, 2016), improving air quality through absorbing and shielding from particulates (Nowak et al., 2006; Hartig et al., 2014), and preventing heat stress by providing shade (Lee et al., 2016). A further ecosystem service is the proposed ability of biodiverse urban green spaces to improve psychological well-being (Sandifer et al., 2015). Such restorative ecosystem services provide one of many arguments for biodiversity conservation (Sandifer et al., 2015). In the United Kingdom, over 80% of the population dwells in urban areas, but along with considerable benefits to health, economies, and education, urbanization has brought great challenges for both social and natural systems (Rook, 2013). Mental health problems affect at least 1 in 6 people in the United Kingdom (Faculty of Public Health, 2010), with an estimated cost to the United Kingdom economy of $105 billion a year, and rising (Mental Health Taskforce, 2016; WHO, 2016). The improvement and expansion of green space has been proposed as a tool for increasing both ecological and psychological well-being in urban environments (Dean et al., 2011), with the Faculty of Public Health claiming that: “safe, green spaces may be as effective as prescription drugs in treating some forms of mental illness” (2010; p. 2). Indeed, 34 English conservation NGO’s have lobbied for 1% of all health spending to be invested in nature-based solutions by 2018 (Response for Nature Partnership, 2015).

The value of green space for psychological well-being has gained increasing academic attention (Sandifer et al., 2015). From the holistic intervention of wilderness therapy (Norton, 2010) to a view of greenery out of the window (Herzele and De Vries, 2012; Honold et al., 2016), many aspects of interaction with green space are being recognized as effective tools for improving well-being. Kaplan and Kaplan (1989) proposed that access to green spaces in urban environments allows the mental fatigue of modern life to be countered by “psychological restoration.” Proximity to green space has been found to improve psychological health through: decreasing cortisol levels (Roe et al., 2013), acting as a buffer to stressful life events (Van Den Berg et al., 2010), increasing social cohesion (Hartig et al., 2014; Gonzalez and Kirkevold, 2016), decreasing maternal depression (Mceachan et al., 2016), and increasing general psychological well-being (Annerstedt et al., 2012; Triguero-Mas et al., 2015). As well as proximity to green space, the “dose” of green space exposure has been shown to impact the benefits gained (Shanahan et al., 2016), with Cox et al. (2017a) finding that 27% of depression cases could be prevented by spending 5 h or more a week in a garden. However, despite many individual studies demonstrating evidence of a mental health benefit the quality of evidence is often poor and general trends from systematic reviews are weak (Gascon et al., 2015). A major issue is the methodological quality of previous studies, which have led to inconsistent results (Lee and Maheswaran, 2011; Hartig et al., 2014).

Our understanding of the links between green space and psychological well-being may be confounded by other factors that influence the value of green spaces for psychological restoration (Nordh and Ostby, 2013; Taylor and Hochuli, 2015). Specifically, the relationship between green space and health has often been investigated through proximity of people to green areas, and neighborhood “greenness” through normalized difference vegetation index (NDVI) values (Ekkel and De Vries, 2017). Francis et al. (2012), however, contend that quality is as important for mental health as quantity, and that research focusing only on quantity is not sufficient to inform policy on public health, or indeed to aid biodiversity conservation (Dean et al., 2011; Taylor and Hochuli, 2015). Green space quality can be quantified from the perspective of the human user through measurement of furniture, management, and cultural cues that can make green spaces feel safe and accessible (Nassauer, 2004; Roberts et al., 2018), and therefore potentially more conducive to relaxation and psychological restoration (Kazmierczak, 2013; Nordh and Ostby, 2013) alongside many other benefits (WHO, 2016). This may include: lighting, adequate seating, signs, indications of management such as cut grass, and lack of graffiti and litter. Green space quality can also be assessed from the perspective of the ecosystem, by quantifying habitat diversity, species diversity, or ecological functions (Lovell et al., 2014; Sandifer et al., 2015). The discussion over what features of green spaces influence attitudes and benefits is a relatively recent addition to the field (Keniger et al., 2013), and has led to a shift from a discussion of “macro” (Dadvand et al., 2014; Mceachan et al., 2015) to “micro” features, and biodiversity in particular (Schebella et al., 2017). For example, a study of interactions with particular plant species in Berlin parks demonstrated that 26 different species of plant formed the basis for green space use for consumption, decoration, or biodiversity experience (Palliwoda et al., 2017). While experimental laboratory work has suggested that there is little difference between the restorative benefits of very different types of natural scenes (Van Den Berg et al., 2014), there is a strong cross-cultural preference for semi-natural green spaces as opposed to more formal parks (Žlender and Ward Thompson, 2017).

What is unclear is whether the presence of site facilities conflicts with high biodiversity (e.g., a trade-off in space use for amenity vs. natural features), and so for a nuanced view of the benefits of green spaces, the correlations between these two components of quality need to be understood. Indeed, a trade-off between site facilities and ecological function is not inevitable, nor are the two components of green space quality necessarily mutually exclusive. Although urban areas often contain far fewer species than rural areas, they retain the ability to hold endemic and sometimes diverse wildlife populations (Aronson et al., 2014) and urban areas can contain more species than rural areas in some cases (e.g., plant richness peaks at intermediate levels of urbanization Mckinney, 2008), thus urban green spaces are increasingly being seen as important stepping stones for wider biodiversity conservation goals (Dearborn and Kark, 2010; Goddard et al., 2010). This diversity can contribute to the positive experience of park users. Lepczyk et al. (2017) noted that small urban green spaces, such as parks, can be incredibly diverse, depending on their connectedness and their habitat quality (Matthies et al., 2017). However, some large open green spaces can have little ecological value, consisting largely of species-poor amenity grassland. Hence, variation in benefits may result from site level factors beyond simple size considerations, which raises the important (but neglected) point that green space area, habitat cover, and biodiversity are not interchangeable concepts (Lepczyk et al., 2017). Urban planning for public health requires an understanding of how site facilities and biodiversity of green spaces are associated with the restorative benefit derived from those spaces.

In addition to research over access to natural spaces in general, there is a considerable body of work on the specific aspects of nature that confer benefits. In particular, it has long been understood that the experience of biodiversity and other aspects of the natural world can act through psychological and psychophysiological mechanisms to enhance well-being (Wilson, 1984; Roszak et al., 1995). However, research that has attempted to investigate the link between biodiversity in green spaces and psychological well-being has produced mixed results (Lovell et al., 2014). Fuller et al. (2007) found a correlation between species richness and psychological benefit in parks in Sheffield, whereby the benefits gained from visiting green space were higher with both higher bird and higher plant diversity. Similarly, Cox et al. (2017b) found that both vegetation cover and afternoon bird abundance in urban areas reduced the severity of depression, anxiety, and stress. In contrast, Dallimer et al. (2012) found a lack of a consistent relationship between diversity and psychological benefit. Instead, they found that perceived species richness did correlate to well-being, but that perception did not correlate to actual species richness. However, studies focusing on flower meadows have shown that perceived and actual species richness do correlate strongly, and that plant, bird, and butterfly richness were positively associated with well-being (Southon et al., 2018). The difference in the accuracy of public judgments of biodiversity may relate to the greater number of more salient cues to diversity in plants, such as color, vegetation height, and evenness (Southon et al., 2018). Fuller et al. (2007) posited that some of the benefit from increased biodiversity might be manifested through environmental cues such as habitat heterogeneity, finding that the number of habitats also correlated to well-being measures. Similarly, tree cover has been shown to be correlated with psychological well-being, with the suggestion that tree cover is a proxy for perceived “naturalness” (Dallimer et al., 2012). The link between biodiversity of green spaces (however, measured) and psychological well-being remains unclear, and few studies have also attempted to incorporate an analysis of non-biological (site facilities) quality.

### Research Aims and Objectives

The current literature suggests a strong, positive relationship between urban green spaces and psychological well-being, but the mechanisms are unclear. Our study aims to fill an important gap in the literature by answering the following research questions:

(1) What are the relationships between site facilities and biodiversity within urban parks set in a multicultural deprived urban area?
(2) How are the site facilities and biodiversity of parks related to psychological restoration?
(3) Do relationships between the features of parks (site facilities or biodiversity) and psychological restoration vary amongst population subgroups (for example, by ethnicity, age, or gender)?

Through the rest of the paper, the following terms are used: “biodiversity” – an umbrella term for biological diversity than encompasses species richness and the number and diversity of habitats; “species richness” – the number of species found in a particular area; “site facilities” – the non-natural objects found within the green spaces (e.g., benches, dustbins, and lighting) and the quality of those amenities (e.g., presence of litter and graffiti).

## Materials and Methods

**FIGURE 1:**
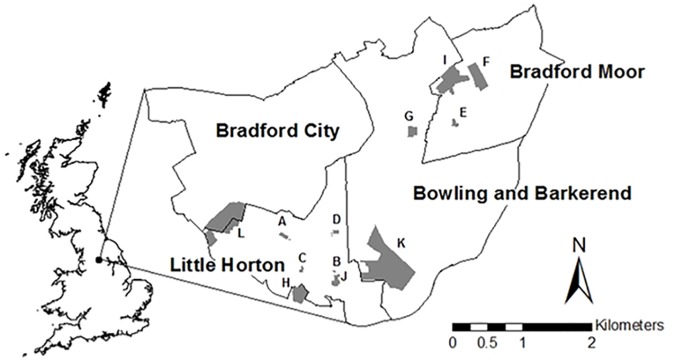
A map of the locations of the parks selected for study across the Better Start Bradford area, denoted by letters.

The study was undertaken in the city of Bradford, the fifth largest Metropolitan district council in England with a population of 534,300 and was further located within the Better Start Bradford programme area. Better Start Bradford is a Big Lottery funded project set within three most deprived wards of the city (Little Horton, Bowling and Barkerend, and Bradford Moor; total population of 63,400; Dickerson et al., 2016). The programme aims to improve health outcomes for some of the most deprived families in the country and includes a focus on improving local green spaces within the area to promote their use^1^. We restricted our work to formal green spaces (defined as sites that are managed, with a structured path network, and an organized layout) which are managed by a single local government department and which are relatively homogeneous in structure and purpose. Google Earth was used to locate potential sites, and site visits were used to select those parks that met the requirements of: constant and full public access, fenced areas in which children could play, and benches as a minimum of park furniture. We selected these minimum requirements to constrain the variation in green space structure to a range of green space types that might be more highly used by the general public. A total of 12 green space sites (parks and recreation grounds) that met these requirements were located within the Better Start Bradford wards, or had a boundary with the area (see Figure 1) and so all work was done in those areas. As even very small parks have been shown to have restoration potential (Krekel et al., 2016), no area constraints were used in selecting sites, however, area was considered in the analysis.

Before describing the methods, it is worth noting that there are potential issues with the definition of “nature” as it relates to the ecological and psychological sciences. Urban ecology has traditionally considered “natural” and “urban” spaces as being spatially separated, while urban spaces can contain biodiversity that varies by degrees in its similarity to that of natural landscapes (Mckinney, 2008). On the other hand, psychological or social definitions of nature experiences tend to include all organisms irrespective of anthropogenic impact alongside socio-cultural context and the changes that occur within the individual as a result of the experience (Clayton et al., 2017). It is worth noting that biodiversity could also incorporate some aspects of ecological processes or functions to complement the biodiversity and habitat variables that we describe above. For example, the quality of an ecosystem could involve the integrity of processes such as nutrient cycling or carbon sequestration, or structural features such as the complexity or functional redundancy of ecological networks. In addition, biodiversity should also not be seen necessarily as a proxy for “naturalness,” as unnatural but biodiverse areas (e.g., floristically diverse horticultural landscapes) could provide considerable restorative benefit. The methods described above consider all biodiversity (whether “natural” or “anthropogenic” in origin) as a single category, and so the resulting measures of biodiversity should not be seen as equivalent to “naturalness” or “wildness.” Hence, the methods represent the common public perception of “nature” as the “living world,” as opposed to ecologically natural communities of organisms.

### Study 1: Green Space Assessments

The site facilities within green spaces were measured using a prototype version of the Natural Environment Scoring Tool (NEST, Gidlow et al., 2018), adapted from the Neighborhood Green Space Tool (Gidlow et al., 2012), that assesses: access, recreational facilities, amenities, natural features, and incivilities. “Access” refers to entrance points and paths, and “recreational facilities” to availability of features such as playgrounds and sports courts, as well as space for physical activity including walking. “Amenities” relates to the placement of sufficient seating, bins, and lighting, “natural features” refers to the maintenance and aesthetics of features such as grass and shrubs, and “incivilities” measures anti-social behavior such as littering, graffiti, and signs of alcohol and drug use. As the tool is a subjective, qualitative scoring measure, two researchers independently assessed each park, and then discussed the results to produce an agreed total score, using Gidlow et al.’s (2018) scoring and weighting system.

While site facilities incorporate a component of natural features (6 out of 47 items on the NEST), we defined a separate measure of biodiversity based specifically on species richness and habitat structure. At each green space, habitats were mapped by identifying the locations and extents of different habitat types. Habitat types included: anthropogenic surfaces (such as playgrounds, sports courts, buildings, and paths), tree cover, amenity grassland, scrub/shrubs, unmown grassland, rough grassland, and waterbodies. Habitat maps were then drawn up for each park using ArcGISv10.4.1, in order to calculate the total area, and the area covered by each habitat type. Tree cover was estimated by drawing polygons around canopy cover from satellite images and habitat diversity was then calculated from the percentage cover of different habitats using Shannon’s diversity index (Dallimer et al., 2012).

Biodiversity surveys were undertaken in such a way that they were representative of the diversity that an individual might experience on a visit to the park. In order to survey the plant species richness at each site, 5 1 m × 1 m quadrats were sampled in each habitat type, or until all of a given habitat was sampled. In woodland, quadrats were 10 m × 10 m, and were adapted to fit the habitat area where this was not possible. Species richness was the total number of species found in all of the quadrats at each green space. Animal species that are likely to be noticed on an everyday visit to parks in the summer (birds, butterflies, and bees) were also surveyed. To conduct bee and butterfly surveys, transects were walked across each site that encompassed all habitat types (Fuller et al., 2007). Any individual butterflies and bees that were seen within 2.5 m on either side of the route and less than 5 m in front of the observer were recorded (Pollard et al., 1986). At the same time, any birds that were seen or heard within the green space area were also recorded (Fuller et al., 2007). The transects were walked twice, once in June and once in July at least a week apart, in suitable weather conditions, with temperatures of at least 13°C, under 50% cloud cover, and wind speeds of no more than 5 on the Beaufort Scale (Carvell et al., 2016). Species richness was the total number of species encountered on the two visits. Although avian species richness is highest in the 3 h after dawn, this is not the time during which people are most likely to utilize green space, and Cox et al. (2017b) found that psychological benefit was correlated to afternoon bird occurrence. Therefore, the surveys were all conducted between the hours of 10am and 5pm in an attempt to sample the species richness that might be experienced on a typical visit to the green space in the summer. This suite of ecological variables incorporate not only taxonomic diversity but also aspects of ecological processes and ecosystem functions such as pollination (bee and butterfly diversity) and carbon sequestration (tree diversity and cover).

### Study 2: Restorative Benefit Surveys

#### Measures

The potential for psychological restoration was measured through four 5-point Likert scale questions used by Nordh et al. (2009) in their photo-elicitation study about pocket parks in Scandinavian cities, and were based on the Attention Restoration Theory (ART) of Kaplan and Kaplan (1989) and the 21 point ART questions developed by Hartig et al. (1997). Kaplan and Kaplan’s (1989) theory is based on four characteristics of green spaces that influence psychological well-being: ‘fascination’ whereby attention is grabbed by elements such as biodiversity through exploration of the site; “being away” as the ability to be psychologically removed from the strains of everyday life; “extent” refers to the order and coherence of a site; and “compatibility” explores how well a green space matches what an individual wishes to do on site. Nordh et al. (2009) focused on overall restoration, being away and fascination from Hartig et al.’s (1997) ART scale, and general preference for a space. That study validated a reduced form of the 21 item ART survey which used the four questions on being away, fascination, likelihood of restoration, and preference in order to reduce the burden on participants (Nordh et al., 2009). For each question, participants were asked to rate the park from 1 to 5, where 1 is strongly disagree, 3 is neutral, and 5 is strongly agree. Answers to the four restoration questions were averaged to give a general score for restorative benefit for each participant. As well as psychological restoration potential questions, all survey participants were asked to complete six 5-point Likert questions about “connectedness to nature” (a measure of the emotional connection or “oneness” with the natural world) that were a short form of Mayer and Frantz’s (2004) scale, developed by Nisbet and Zelenski (2013), to assess if connection to nature had any bearing on restoration potential. Answers to these six questions were also averaged to give a general score of connectedness to nature for each participant. Participants were also asked about their use of the park, such as the activities that they undertake, and how often they visit (see Supplementary Information for the full questionnaire). Demographic data on age, gender, and ethnicity were also collected.

#### Participants

All adults entering or leaving the park during the time of the audit were considered to be eligible to take part. In the busier parks, every third person was approached. However, in the less visited parks, to ensure that adequate sample sizes were reached, every person entering the park was approached. All participants completed the surveys alone, with people in pairs surveyed separately by different researchers, and only one person per group of >2 approached to reduce non-independence of responses. Children were not approached to avoid concerns over vulnerability and to allow the development of a single, age-appropriate survey. Participant demographics were compared against 2011 United Kingdom census data for the three wards (Bowling and Barkerend, Bradford Moor, and Little Horton) to evaluate the representativeness of the sample.

#### Procedure

Face to face surveys were conducted in English by two interviewers in July and August 2017 at the entrance to each of the 12 parks. At least 12 person-hours of survey effort was undertaken in each park at similar times of day in order to obtain a comparable sample of park users for each site. After taking informed consent, the survey took approximately 10 min to complete. Ethical approval was given from the Faculty of Biological Sciences Ethics Committee (reference: LTSBIO-004).

### Statistical Analysis

All statistical analysis was conducted in R (R Core Team, 2015), with the lme4 (Bates et al., 2015), car (Fox and Weisberg, 2011), MuMIn (Barton, 2016), and LMERConvenienceFunctions (Tremblay and Ransijn, 2015) packages. In Study 1, all variables approximated a normal distribution in a Shapiro–Wilk normality test, and so Pearson correlations were used to test for associations between different variables. In Study 2, we calculated a single value for restorative benefit and connectedness to nature for each participant by averaging the scores for the component questions (four questions for restorative benefit and six questions for connectedness to nature). Although Likert scale data is ordinal, averages taken across the scale are often treated as continuous and subjected to parametric analysis (e.g., Fuller et al., 2007; Dallimer et al., 2012), and discussion in the statistical literature suggests that there is a strong basis for doing so, as long as the model assumptions are met (Harpe, 2015). For all of the tested linear models, the residuals were inspected to ensure that the assumptions of homogeneity of variance and normality were not violated.

In Study 2, analysis of restorative benefit was done in a two-tiered approach, whereby park level relationships (*n* = 12) were first considered by using the average restorative benefit across all participants to give a value for each park in linear regression models. This park-level analysis accounted for the potential pseudoreplication that may have arisen from having participants surveyed from the same small set of parks. However, the park-level analysis also lacked statistical power due to the focus on a relatively small number of sites. As a result, a second, complementary analysis was also conducted using the individual-level data (*n* = 128). This individual-level analysis used linear mixed effects models with the park as the random effect. In this way, we examined patterns across individual participants while accounting for the fact that they experience different park environments. In both cases, we use a model selection approach based on Akaike’s information criterion (AIC). First, we began with a full model containing all variables of interest (habitat diversity, number of trees, species richness of birds, plants, bees/butterflies, habitat number, and site facilities). Since there was a strong probability that some of these predictors would be collinear, and therefore would have inflated the estimation of standard errors associated with parameters, we checked for multicollinearity using variance inflation factors (VIFs). Where collinearity was identified in the ecological variables (VIF ≥ 5, Akinwande et al., 2015) we used a principal component analysis (PCA) to summarize those variables. The PCA produced orthogonal principal components that explain different dimensions of a higher-order dataset and can be included as predictors in place of the collinear variables. Next, we constructed a set of models containing different combinations of predictors. Since there were no strong *a priori* expectations of which parameter combinations might provide the strongest fit, the full models for both studies (green space quality and restorative benefit), and both levels of analysis (park-level and individual-level) were then subjected to a comparison of all possible fixed effect combinations. The mixed models were fitted by maximum likelihood to allow model comparison. This process generated a large set of models which were then compared using AIC to find the best-fit model in each analysis. Where multiple models exhibited similar AIC scores (ΔAIC < 2) we used model averaging to calculate parameter estimates that incorporated information from each of those top models, weighted by their goodness of fit (Grueber et al., 2011).

## Results

### Study 1: Assessment of Park Quality

Across all of the sites, there was substantial variation within the park quality metrics (see Table 1), with both plant (16–100 species) and bird (4–21 species) richness varying by a factor of five. From the ecological surveys, the most abundant and constant herbaceous species were: perennial ryegrass (*Lolium perenne*), annual meadow-grass (*Poa annua*), and white clover (*Trifolium repens*), with silver birch (*Betula pendula*), sycamore (*Acer pseudoplatanus*), and cherry (*Prunus* spp.) the most common woody species. The most common bird species were the: feral pigeon (*Columba livia domestica*), blackbird (*Turdus merula*), and house sparrow (*Passer domesticus*). Of the bees and butterflies, the most often encountered were the: small white butterfly *(Pieris rapae*), buff-tailed bumblebee (*Bombus terrestris*), and tree bumble-bee (*Bombus hypnorum*).

**Table 1:** Site level characteristics, including ecological and site facilities characteristics across the 12 studied parks.

Park quality variables	Mean	*SD*	Min, Max
Site facilities (Natural Environment Scoring Tool, 0–100)	52.60	9.17	35, 67
Plant richness (number of species)	47.42	26.77	16, 100
Bird richness (number of species)	11.50	6.43	4, 21
Bee/butterfly richness (number of species)	7.00	3.81	0, 12
Habitat diversity (Shannon’s Diversity Index)	1.09	0.23	0.77, 1.51
Habitat number	4.83	1.12	3, 7
Tree cover (%)	19.09	10.30	6.47, 41.85

**FIGURE 2:**
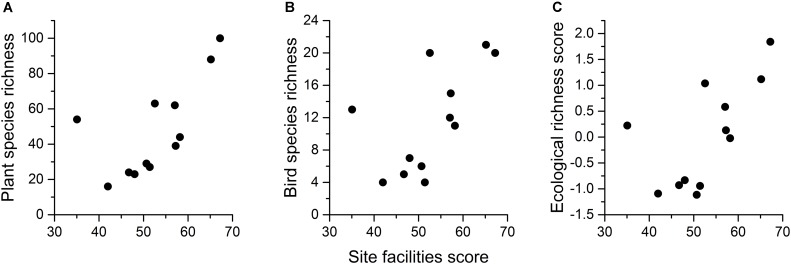
The relationship between site facilities and **(A)** plant species richness, **(B)** bird species richness, and **(C)** the ecological richness score based on a combination of biodiversity-related factors.

Across all 12 sites, the species richness of different taxa was correlated between plants and birds (*r* = 0.901, *p* < 0.001), between bees/butterflies and birds (*r* = 0.637, *p* = 0.026), and also between plants and bees/butterflies (*r* = 0.658, *p* = 0.020). Both plant and bird species richness were correlated with habitat number (*r* = 0.828, *p* = 0.001; *r* = 0.799, *p* = 0.002, respectively), but no diversity or richness variables showed significant correlation with tree cover or habitat diversity. Site facilities (from the Natural Environment Scoring Tool) correlated only with plant (*r* = 0.671, *p* = 0.017), and bird richness (*r* = 0.602, *p* = 0.038) (Figure 2).

Land area varied greatly across the parks, with a range of 0.14–32.56 hectares, and all variables except butterfly and bee richness, habitat diversity, and tree cover significantly correlated to the log_10_ area of the parks (Table 2). For site facilities there was also a strong positive effect of area. Due to the correlations of park variables with area, in subsequent analysis, a model with area as a predictor was run to explore the relationship of area with restorative benefit (Fuller et al., 2007).

**Table 2:** Pearson correlation results between the park variables and park area.

Correlations with log_ 10_ area	*N*	*r*	*P*
Site facilities	**12**	**0.662**	**0.019**
Plant species richness	**12**	**0.805**	**0.002**
Bird species richness	**12**	**0.917**	**<0.001**
Bee/butterfly species richness	12	0.461	0.132
Tree cover	12	0.515	0.087
Habitat diversity	12	0.037	0.908
Habitat number	**12**	**0.647**	**0.023**

*Significant correlations (p < 0.05) are highlighted in bold.*

### Study 2: Restorative Benefit Surveys

Participant demographics were compared against 2011 census data for the three wards in which the parks were located (Figure 1). Country of birth was very similar between survey respondents (65.9% United Kingdom born, 26.4% South Asia born) and the local population (66.2% United Kingdom, 24.0% South Asia), as was the marital status (52.7% married in survey, 49.3% married in census). The survey included a greater percentage of female respondents than the local population (54.7%, compared to 49.8%) and tended to include older individuals (49.6% were aged 46 or older in the survey compared to 18.4% being aged 50 or older in the census). These results suggest that the survey respondents are broadly representative of the local population in terms of race, gender and ethnicity, but that older people were over-represented. Analysis of variance (ANOVA) showed no significant differences in restorative benefit between sexes (*F*_1,127_ = 2.208, *p* = 0.140), United Kingdom-born or foreign born (*F*_1,127_ = 0.341, *p* = 0.560), ethnicities (*F*_1,127_ = 0.302, *p* = 0.584) or ages (*F*_1,127_ = 2.468, *p* = 0.119).

At one site, only one survey was completed during 6 h of interviewing effort due to low use of the space and so that site is excluded from the following analysis. For the remaining 11 sites, a total of 128 participants completed the survey (mean 11.6 per site, range: 5–21) and the participant demographics are presented in Table 3. During survey periods, 63% of people who were approached agreed to take part in the surveys. The mean restorative benefit (averaging four 5-point Likert scale questions where higher numbers indicate greater agreement with statements about restorative benefit) across the 11 sites was 3.635 (SE = 0.094), indicating a general perception of positive restorative benefit. There was a significant difference in the restorative benefit reported by individuals among the 12 sites based on individual-level data (*F*_10,116_ = 3.468, *p* = 0.001).

**Table 3:** Demographic characteristics of survey participants.

Characteristics		% Participants (*n* = 128)
Gender	Male	54
	Female	46
	Other	0
Age	18–25	9
	26–35	19
	36–45	23
	46–55	19
	56–75	26
	76+	4
Ethnicity	Pakistani	40
	White British	36
	Indian	5
	Eastern-European	5
	British Pakistani	4
	Bangladeshi	3

In order to explore the relationships between ecological quality and site facilities and restorative benefit we first tested for collinearity in the predictor variables, which would inflate the SE of parameter estimates. VIF scores were calculated from a full model of all of the variables across the 11 green spaces used in the *in situ* study and showed that four of the biodiversity-related variables (plant, bird, and bee/butterfly species richness, and habitat number) all showed significant collinearity. To account for this collinearity, a new variable, hereafter called “ecological richness score,” was derived from the first principal component of a principal components analysis using the four biodiversity variables. The ecological richness score accounted for 84% of the variance in the four component biodiversity variables and, once this factor was used, the VIF values for terms in the full model were below 3 for all variables.

**FIGURE 3:**
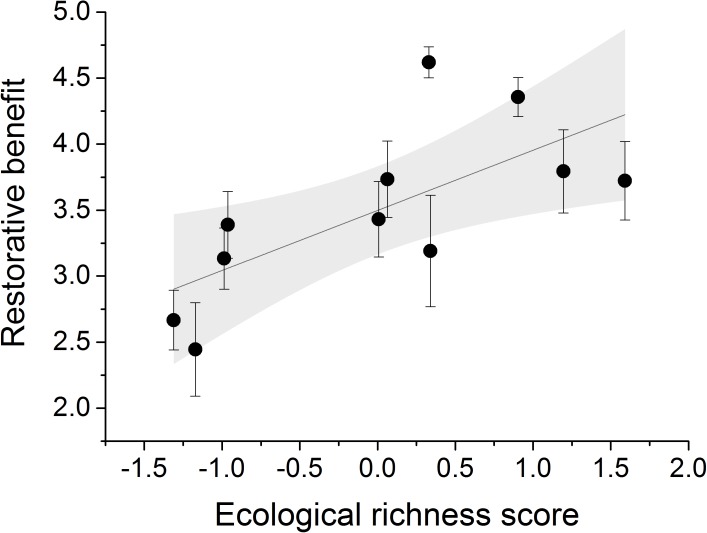
The relationship between the ecological richness score (a composite score based on a combination of plant diversity, bird diversity, bee/butterfly diversity, and habitat number) and the mean restorative benefit of each park. Shaded area represents the 95% confidence band of the linear regression line.

Model selection using park-level data to explain variation in the restorative benefit reported by participants produced two highly supported models (where ΔAICc < 2 of the top model, Table 4). The top model contained only the ecological richness score and the second model contained the ecological richness score and tree cover (Table 5). Only the ecological richness score featured in both models and the model with only the ecological richness score had a model weight of 0.480 and explained 43% of the variance in restoration (*F*_1,9_ = 8.658, *p* = 0.016, *R*^2^ = 0.434, Figure 3).

**Table 4:** The models selected for model averaging where ΔAICc < 2 of the top models.

Model	*df*	AICc	ΔAICc	*w*_i_
**Park-level models**				
Ecological richness score	3	22.72	0.00	0.63
Ecological richness score, tree cover	4	24.56	1.84	0.25
**Individual-level models**				
Ecological richness score, tree cover	5	370.58	0.00	0.20
Ecological richness score, connection to nature, tree cover	6	371.08	0.50	0.20
Ecological richness score	4	371.43	0.85	0.20
Ecological richness score, habitat diversity	5	371.54	0.96	0.13
Ecological richness score, connection to nature	5	371.88	1.30	0.11
Ecological richness score, connection to nature, habitat diversity	6	372.10	1.52	0.10
Ecological richness score, habitat diversity, tree cover	6	372.10	1.53	0.10
Ecological richness score, gender, tree cover	6	372.49	1.91	0.08

*The null models had an AICc of 26.20 and 376.48 and a ΔAICc of 3.49 and 5.90 for the park- and individual-level models, respectively.*

**Table 5:** Averagemodels for park-level and individual-level model selection explaining variation in restorative benefit based on models in Table 4.

Variable	Importance	Coefficient	SE	*Z*	*P*
**Park-level models**					
Ecological richness score	**2/2**	**0.497**	**0.171**	**2.552**	**0.011**
Tree cover	1/2	-0.026	0.015	1.447	0.148
**Individual-level models**					
Ecological richness score	**8/8**	**0.485**	**0.149**	**3.214**	**0.001**
Tree cover	4/8	-0.011	0.014	0.814	0.416
Habitat diversity	3/8	-0.221	0.452	0.488	0.625
Connection to nature	3/8	-0.043	0.094	0.454	0.650
Gender (female vs. male)	1/8	-0.008	0.059	0.139	0.900

*Importance is defined as the fraction of top models (ΔAICc < 2) in which each term is found. Significant parameters are highlighted in bold.*

To evaluate the roles of different predictors of restorative benefit at the level of individual participants, mixed effects models were used to assess the individual level data with site as a random factor. The four biodiversity variables were collinear again, as in the park-level analysis, so the ecological richness score was calculated using PCA as above for this dataset. A full model was created using all variables (park-level data: site facilities, ecological richness score, tree number, habitat diversity; plus individual-level data: ethnicity, age, gender, connectedness to nature) and all possible variable combinations were compared using AIC, then model averaging was used with the model set that was within ΔAICc < 2 of the top model (Table 4). There were not enough individual survey results that included combinations of ethnicity, gender, and age groups to enable all three terms to be considered in the same model. Instead, each of those three variables was used in a separate model with the rest of the terms. Neither ethnicity nor age, however, appeared in the top models, and so only gender appears in the top model set (Table 4). The set of top models indicated that ecological richness score had by far the greatest model importance, appearing in all 16 of the top models (Table 4), as in the park-level analysis. To answer our second research question, restorative benefits perceived from parks were principally predicted by biodiversity, while site facilities did not feature in any of the top models (Table 5). Although connection to nature features in some of the top models, the effect sizes attributable to connection to nature were negligible (Table 5). Finally, in answer to our third research question, we find no evidence of the contribution of ethnicity or age to explaining variation in restorative benefit. While gender appears in some of the top models, the contribution of gender to the statistical model is negligible (Tables 4, 5). Hence, our findings suggest that restorative benefit is independent of demographic characteristics.

## Discussion

We demonstrate that site facilities and ecological (i.e., biodiversity and habitats) quality of parks are positively correlated, suggesting not only that there is no trade-off but that higher quality parks have a function both for amenity and biodiversity. Furthermore, there are strong differences in the restorative benefits obtained from different parks. However, when the associations between different aspects of quality and restorative benefit are compared in models of both parks (*n* = 11) and individuals (*n* = 128), the restorative benefit of the parks appears to be predicted principally by biodiversity rather than site facilities. When different demographic groups are compared, the benefits accrued in terms of restoration are independent of age, gender, and ethnicity. These results add to the growing evidence for an important role of biodiversity in driving the ecosystem services that can be derived from urban landscapes.

The major strength of this study in comparison with the existing literature was the consideration of both site facilities and biodiversity in exploring the benefits of green space. Previous studies have largely only considered site facilities, when biodiversity and habitat measures can make a difference to how people use and interact with green spaces (Nordh and Ostby, 2013; Roberts et al., 2018). In addition, previous studies have focussed on park level data (Fuller et al., 2007; Dallimer et al., 2012), but here the use of mixed models allowed individual level data to be analyzed to explore potential differences in restorative benefit with age, gender, ethnicity, and connection to nature. Restorative benefit and well-being have been shown to be correlated to “natural features” such as a high vegetation cover and plant/flower abundance (Nordh and Ostby, 2013), and plant richness, bird richness, and habitat diversity (Fuller et al., 2007; Southon et al., 2018). However, other studies suggest that the effect is not a direct benefit from biodiversity but is influenced by how biodiverse a person believes that environment to be (Dallimer et al., 2012). Our data support the link between biodiversity and well-being, but not between well-being and connectedness to nature that is commonly reported (Capaldi et al., 2014). One possible explanation could be the malleability of connectedness to nature as a concept. Nature connectedness can be influenced either by heightening self-awareness (Frantz et al., 2005) or by increasing exposure to natural places (Schultz and Tabanico, 2007). It is possible that urban populations have a relative low and similar degrees of connectedness to nature based on sporadic contact with natural places, as both connectedness and positive benefits can be enhanced through exposure to nature (Mayer et al., 2009). While previous studies have demonstrated links between connectedness to nature and well-being, our study was more about the evaluations of place, rather than of self. Hence, a direct relationship between perceived restorative benefit and nature connectedness may not be as intuitive as it first appears.

There were no differences discovered in the restorative benefits of green space between ethnic groups, in contrast to some existing literature (e.g., Dadvand et al., 2014). Access to green space only correlates with health outcomes in minority ethnicities with very poor health, but not with ethnic groups that enjoy better health (Roe et al., 2016) and so we might expect to see a difference between ethnicities. There are two potential explanations for this observation. The first is that our area of study is a relatively impoverished region of Bradford and so all ethnicities may have the same starting poor health. Second, our study population was not local residents for whom benefits from local green space might be influenced by access, cultural differences, economic status, mobility, or health problems, but the park users themselves. It could be that once the different ethnicities access the green space the differences in health outcomes are not significant, in contrast to the results of epidemiological studies that incorporate many barriers to access.

We expected that we would find an effect of age and connection to nature on well-being benefits, as has been described before (Luck et al., 2011), but neither exhibited a significant effect. It is possible that our self-selected sample of individuals – who had already made the choice to engage with the green space (by virtue of conducting surveys within those green spaces) – may exclude those individuals who are less connected to nature, and therefore have less to benefit from increased biodiversity (Mayer and Frantz, 2004). Children have been shown to exhibit no preference for biodiverse or wilder areas, even when they have access to those places (Hand et al., 2017), but only adults were included here and those adults tended to be older than the average for the area. A broader age range may have revealed underlying age-related patterns more clearly, and adult preferences do seem to show a positive association between perceptions of attractiveness and ecological diversity (Davis et al., 2016).

The move toward, and growing evidence base for, nature-based solutions to urban problems may facilitate secondary well-being benefits from new nature-based infrastructure (Keesstra et al., 2018). Nature-based solutions are typically defined as “…*actions to protect, sustainably manage and restore natural or modified ecosystems, which address societal challenges (e.g., climate change, food and water security or natural disasters) effectively and adaptively, while simultaneously providing human well-being and biodiversity benefits*” (Cohen-Shacham et al., 2016). These definitions seem to limit health-related outcomes to ancillary or secondary benefits of solutions that focus on largely engineering-based problems. However, there is clearly a strong need for nature-based solutions that have health improvement as a key primary outcome, and this could be achieved through the enhancement (in extent and quality) of urban green and blue spaces. It is worth noting that closer inspection of green space types has shown that other ecosystem services such as air purification and climate regulation also vary between different types of green space (Vieira et al., 2018). There are obvious implications for general urban green space management and planning. In an urbanizing world where mental illness is not being reduced despite substantial increases in the investment in treatment (Jorm et al., 2017), the central tenet of urban planning must be human health and well-being (Barton et al., 2009), and this process must necessarily include a consideration of biodiversity (Sandifer et al., 2015). A survey of green space managers in America showed that they feel that there is a movement toward managing for ecosystem services (Young, 2010), such as improving human well-being. Similar patterns are seen among Swedish green space managers, who report local targets for green spaces in terms of stormwater management, education, and health alongside biodiversity (Randrup et al., 2017). Broader trends in “urban greening” suggest that increasing nature in cities is associated with a number of outcomes, including biodiversity and health, across the United States, Canada, and Western Europe (Anguelovski et al., 2018). Yet studies that have considered all green spaces as equally valuable have not been adequate to inform policy on improving human health and would likely have no impact on improving urban biodiversity (Dean et al., 2011).

The number of studies considering associations between different aspects of well-being and a broad range of green space variables is small and so it is premature to make policy recommendations. However, there are potentially substantial, cost-effective health gains to be made should policymakers incorporate green space enhancement into health care budgets. Funding associated with the maintenance of urban green spaces has been reduced for 92% of park managers in the United Kingdom from 2012 to 2015 (Heritage Lottery Fund, 2016), while costs of mental health treatment and impacts continue to increase to over $100bn (Department of Health, 2011). Results such as ours provide further provisional support for the prescribing of green space as a cost-effective investment for mental health, in addition to the current park prescriptions for physiological health. With green space being increasingly proposed as a tool to aid psychological well-being, the addition of increasing biodiversity as a management strategy (e.g., Taylor and Hochuli, 2015) also has great implications for improving urban conservation and restoration of biodiverse habitats. Urban planning that includes many connected, high quality green spaces has the potential to provide major improvements to the ability of urban areas to hold diversity and connect surrounding areas (Goddard et al., 2010; Lepczyk et al., 2017). Subsequent work should examine the impacts of budgetary limitations on the maintenance of urban green spaces and the trends in ecosystem services that are derived from them. Visitors to parks often view both naturalness and neatness as high priorities for green spaces, which complicates management (Ngiam et al., 2017). Neatness also factors into issues of safety, which are often also antagonistic to biodiversity and naturalness (Schroeder and Anderson, 1984). However, it has been suggested that rather than “de-vegetating” to make spaces safer through the elimination of hiding places, it would be better to “re-people” spaces through the creation of social events in those spaces (Gobster and Westphal, 2004).

The current study has some limitations. It was based within a multicultural deprived urban area, and the extent to which the findings translate to more affluent areas needs be demonstrated. There is a lack of evidence concerning the precise mechanisms by which ecological or biological park parameters (e.g., species richness) are perceived by and influence people, and it is possible that our suite of ecological measurements excludes some parameters that are relevant. For example, area itself may have a direct impact on restorative benefit via a sense of isolation, or might enhance opportunities for exercise which, in turn, improve well-being (Berman et al., 2012; Aspinall et al., 2015). Perceived naturalness (which is likely linked to area) has been shown to interact with perceived restorativeness to enhance positive outcomes from green space exercise (Marselle et al., 2015). Acoustic stimuli have also been shown to be important in stress reduction (Alvarsson et al., 2010; Annerstedt et al., 2013), but soundscapes were not monitored in the green spaces in this study. Due to resource limitations we were unable to include participants who did not speak English, and so the results may underestimate ethnic differences if those potential participants with larger cultural differences were unable or unwilling to take part. Participants completed surveys *in situ* and their perceptions of the psychological restoration of parks may differ from non-park users.

Our survey sample was broadly representative of the local resident population based on census data, apart from an over-representation of older individuals. Age is known to be one of a range of factors that influence self-reported measures of well-being in national surveys (Steptoe et al., 2015) but was not associated with well-being in this study. More generally, subjective measures of well-being have been shown to be associated with lower mortality and have been advocated for inclusion in national statistics for social and economic progress (Stiglitz et al., 2009). There is a considerable body of instruments that can be used to evaluate subjective well-being in a generic sense, with great variation between those instruments in the conceptual basis and what, exactly, is being measured (Linton et al., 2016). However, our approach uses a focused and well tested survey instrument that, while it is specific to restorative benefits associated with place, has been shown to be consistent and well-grounded in theory (Hartig et al., 1997; Nordh et al., 2009). Despite this, there are considerable opportunities to enhance data collection using objective measures of well-being, such as health outcome data from longitudinal studies (Dadvand et al., 2014; Mceachan et al., 2015) or *in situ* measures of stress (Roe et al., 2013).

## Conclusion

Our paper is one of the first to explore explicitly relationships between objectively assessed biodiversity, site facilities and participant reported assessed of psychological restoration. We found that biodiversity and site facilities were positively correlated within urban parks. However, we found that only biodiversity was related to perceptions of psychological restoration amongst a multi-ethnic group of participants. These findings suggest that urban planners should aim to enhance ecological diversity in urban green spaces. Specifically, there are likely to be secondary benefits from nature based solutions in cities which introduce additional green or blue infrastructure in place on gray infrastructure. However, there are also opportunities for nature based solutions that have health outcomes as a primary aim, such as expanded or increased numbers of parks, planting of trees to minimize urban noise pollution, and enhancement of botanical or floral diversity that seems to be most strongly associated with restorative benefit across studies. Future research is warranted to test the replicability of these emerging findings in other social, geographic and ecological contexts. Beyond epidemiological studies, empirical work is particularly needed to produce a stronger and more persuasive evidence base for policymakers.

## Ethics Statement

This studywas carried out in accordance with the recommendations of the Faculty of Biological Sciences Research Ethics Committee at the University of Leeds. The protocol was approved by the Faculty of Biological Sciences Research Ethics Committee at the University of Leeds (ref: LTSBIO-004). All subjects gave written informed consent in accordance with the Declaration of Helsinki.

## Author Contributions

All authors contributed to the design of the experiment and writing of the manuscript. EW and AH collected the data in the field. EW and CH analyzed the data.

## Conflict of Interest Statement

The authors declare that the research was conducted in the absence of any commercial or financial relationships that could be construed as a potential conflict of interest.

## Footnotes

1 www.betterstartbradford.org.uk
