# Supplementary material for: Not All Green Space Is Created Equal: Biodiversity Predicts Psychological Restorative Benefits From Urban Green Space
Source: Front Psychol. 2018 Nov 27;9:2320. doi: 10.3389/fpsyg.2018.02320 (PMC6277587; doi:10.3389/fpsyg.2018.02320)
Supplement: Supplementary file 1 [file Presentation_1.pdf]

frontiers

Supplementary Material

Not All Green Space Is Created Equal: Biodiversity Predicts Psychological Restorative Benefits From Urban Green Space

Emma Wood, Alice Harsant, Martin Dallimer, Anna Cronin de Chavez, Rosemary R. C. McEachan and Christopher Hassall*

*** Correspondence:** Christopher Hassall: c.hassall@leeds.ac.uk

This appendix contains a copy of the questionnaire used in the in situ park surveys. Note that some questions were exclusively for use in another study, and are highlighted by *.

Demographic questions

Age:

☐ 18-25

☐ 26-35

☐ 36-45

☐ 46-55

☐ 56-75

☐ 76+

Sex:

☐ Female

☐ Male

What best describes your ethnic group or background?

☐ White; English/Welsh/Scottish/Northern Irish/British

☐ White; Irish

☐ Pakistani

☐ Indian

☐ Bangladeshi

☐ White; Polish

☐ White; Slovakian

☐ White; Romanian

☐ White; Czech

☐ Other White

☐ White; Gypsy/Roma or Irish traveller

☐ Chinese

☐ African

☐ Caribbean

☐ Mixed White and Black Caribbean

☐ Mixed White and Black African

☐ Mixed White and Asian

☐ Other ethnic or mixed/multiple ethnic background___________________________________

Place of Birth:

Marital Status:

- ☐ Single
- ☐ Partner
- ☐ Divorced
- ☐ Widowed

Do you have any children?

If so, what are their ages?

Questions relating to use and perceptions of green space

Which local park or greenspace/s do you visit most often?

How often do you visit a local greenspace or park in the spring and summer?

- ☐ every day
- ☐ a few times a week
- ☐ once a week
- ☐ once a month
- ☐ a few times a year
- ☐ never

How often do you visit a local greenspace in the autumn and winter?

- ☐ every day
- ☐ a few times a week
- ☐ once a week
- ☐ once a month
- ☐ a few times a year
- ☐ never

Do you visit the park mainly on the weekend or a weekday?*

- ☐ weekend
- ☐ weekday
- ☐ both

How far do you live from a park or greenspace?*

- ☐ <5 minutes' walk
- ☐ 5-10 minutes
- ☐ 10-15 minutes
- ☐ further

How satisfied are you with the park, and why?*

1 2 3 4 5

What activities do you do or could you do in the local parks?

What activities do or could young children do in the local parks?

Questions relating to the naturalness of green spaces

What wildlife do you see in the local parks? *

	Yes/no	How many species, roughly?
Birds		
Mammals (e.g. squirrels, mice, hedgehogs)		
Butterflies		
Plants (including trees)		
Insects		

Do you have a garden? If so, what size?*

- ☐ small yard
- ☐ smaller than a tennis court
- ☐ larger than a tennis court

If so, what plants and animals do you see there?

Questions on connectedness to nature

If you think the statement is not like you score it a 1, if the statement sounds a lot like you then score a 5 and if you are neutral about the statement score a 3.

	1	2	3	4	5
My ideal vacation spot would be a remote, wilderness area.					
I always think about how my actions affect the environment.					
My connection to nature and the environment is important to me					
I take notice of wildlife wherever I am.					
My relationship to nature is an important part of who I am.					
I feel very connected to all living things and the earth.					

Questions on restorative benefit

If you strongly agree with the statement, score it a 5, if you strongly disagree with the statement, score it a 1, and if you are neutral, score a 3.

	1	2	3	4	5
There is much to explore and discover here.					
This place is a refuge from unwanted distractions.					
I would be able to rest and recover my ability to focus in this environment.					
I like this environment.					
I feel safe in this park.*					

What features in the park make you think you could feel most relaxed?

What features in the park make you think you could not feel relaxed?

What features would you like to see added to a park to make it more relaxing?

Have you heard of Better Start Bradford?*
